# Paradox inflammatory reaction such as appendicitis epiploica and diverticulitis of the sigmoid colon under ongoing immunosuppression after previous liver transplantation (LTx)

**DOI:** 10.1515/iss-2023-0038

**Published:** 2023-09-15

**Authors:** Isabella Trautwein, Manuela Petersen, Christine March, Roland S. Croner, Frank Meyer

**Affiliations:** Department of General, Abdominal, Vascular and Transplant Surgery, Otto von Guericke University Medical School, Magdeburg, Germany; Department of Radiology and Nuclear Medicine, Otto von Guericke University Medical School, Magdeburg, Germany

**Keywords:** intraabdominal inflammatory reaction, appendicitis epiploica, immunosupression, previous liver transplantation

## Abstract

**Objective:**

Inflammatory reactions caused by immunosuppression appear a particular interesting disease due to its very specific and partly unclear etiopathogenesis.

Based on clinical case-specific management experiences and selective references from the literature, the rare case of an acute intraabdominal inflammation as unusual complication or side effect (at the gastrointestinal [GI] tract) of the ongoing immunosuppressive medication using Mycophenolate mofetil and Tacrolimus after previous liver transplantation is to be illustrated.

**Case presentation:**

*Medical history* (*hx*): 1) *Current*: A 68-years old male patient underwent abdominal CT scan because of pain in the left lower abdomen with the suspicious diagnosis of diverticulitis leading to initiation of antibiotic therapy 24 h prior to the transferral to the own hospital for adequate liver transplantation (LTx) follow-up investigation. 2) *Medication* contained Sitagliptin 1 × 100 mg, Omeprazol 1 × 40 mg, Mesalazin 500 mg 3 × 2, Movicol 1 (on demand), Mycophenolate mofetil 2 × 500 mg, Tacrolimus 2 × 1 mg and Hydrochlorothiazid 1 × 2.5 mg. 3) *Additional diagnoses* included arterial hypertension, diabetes mellitus and urinary bladder diverticle. 4) *Previous surgical intervention* profile comprises resection of liver segments IV/V due to HCC (2011), orthotopic liver transplantation because of HCC caused by alcohol-induced liver cirrhosis (2013) and an intervertebral disc operation (2018). *Physical examination* of the abdomen revealed marked tenderness in the lower left quadrant. The abdominal wall was soft and there were no defensive tension and no peritonism. The patient was in good general condition and nutritional status. He was cardiopulmonarily stable and oriented to all qualities. *Diagnostic measures* showed a CRP of 38.0 (normal range, < 5) mg/L and a white blood cell count within normal range. Leading diagnoses were found using abdominal CT scan, which demonstrated an extended diverticulosis and an appendicitis epiploica within the immediate subperitoneal region of the left lower abdomen with an oval fat isodense structure in the region of the sigmoid colon with surrounding inflammatory imbibition and pronounced intestinal wall. *Suspicious diagnosis* was the 1st episode of an uncomplicated diverticulitis of the sigmoid colon associated with an appendicitis epiploica. *Therapeutic approach* was given by conservative therapy with infusion therapy, analgesia as well as inital “n. p. o.” and following initiation of oral nutrition. In addition, calculated antibiotic therapy with Cefuroxime and Clont was initiated. *Clinical course* was uneventful, with discharge on the eighth day of hospital stay with no pathological findings and substantial improvement in clinical and laboratory findings. *Further advice* consisted of clinical and laboratory follow-up control investigations by the family practitioner and nutritional counselling. In addition, a colonoscopy should be performed within four months.

**Conclusions:**

The described case i) is either one of the many side effects of the immunosuppressive medication Mycophenolate mofetil and Tacrolimus listed as “colonic inflammation” and “gastrointestinal inflammation”, respectively, or ii) can be considered an inflammatory response of a susceptible (gastro-)intestinal mucosa or the whole intestinal wall to microbes or microbial particles or agents caused by transplantation-associated immunosuppressive medication.

## Introduction

Inflammatory reactions under ongoing immunosuppression appear to be an interesting pathology due to their particular etiopathogenesis. In general, immunosuppressants interfere very strongly with important inflammatory and anti-inflammatory processes and its interplay in the body, such as the release of prostaglandins and others. Thus, the advantages and disadvantages must be considered carefully before administration in order to achieve a positive outcome. Currently, combinations of several immunosuppressive drugs are used for immunosuppression, which must be given to each patient individually so that the therapeutic aim is achieved, but there is no existing standard combination. To achieve such standard, it is necessary to adequately understand the processes occurring in the body during immunosuppression.

The aim was to i) illustrate – by means of a scientific case report based on patient-specific experiences obtained in the clinical management and selective references from the scientific medical literature – the rare case with an acute intraabdominal inflammatory focus as an unusual complication or adverse effect of the immunosuppressive medication including Mycophenolate mofetil and Tacrolimus after previous orthotopic liver transplantation and ii) discuss the possible etiopathogenetic background.

## Case summary

### Medical history

A 68-year-old male was transferred from a regional hospital for further diagnostic and treatment of abdominal pain in the lower left quadrant. The patient had a medical record of liver resection (segments IV/V) due to hepatocellular carcinoma in 2011. In March 2013, the patient underwent orthotopic liver transplantation (LTx) because of hepatocellular carcinoma caused by alcohol-induced liver cirrhosis. In September 2018, an intervertebral disc operation was performed. Other pre-existing conditions include arterial hypertension, diabetes mellitus and urinary bladder diverticle.

### Medication at admission

**Table:** 

Sitagliptin	100 mg	1 × 1
Omeprazol	40 mg	1 × 1
Mesalazin	500 mg	3 × 2
Movicol		1 (on demand)
Mycophenolate mofetil	500 mg	2 × 1
Tacrolimus	1 mg	2 × 1
Hydrochlorothiazid	2.5 mg	1 × 1

### Physical examination

The patient was in good general condition and nutritional status. He was cardiopulmonarily stable and oriented to all dimensions. The abdominal wall was soft but palpation revealed marked tenderness in the lower left quadrant. There were no defensive tension and no peritonism.

### Diagnostic measures

*Laboratory values* upon admission showed inflammatory signs with a serum CRP level of 38.0 (normal range, < 5) mg/L. The white blood cell count was within normal range.

**Figure 1:**
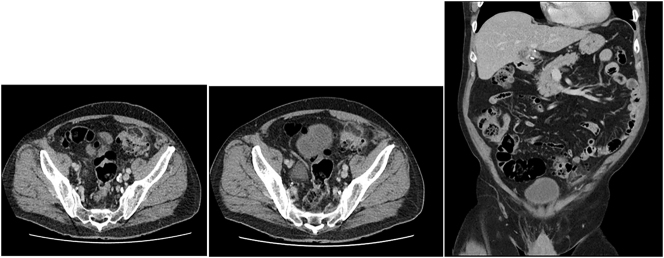
Abdominal CT scans – axial (A and B) and coronal sectional plane (C): Extended diverticulosis and appendicitis epiploicae in the immediate subperitoneal region of the left lower abdomen with an oval fat isodense structure in the region of the sigmoid colon starting from the anterior wall with surrounding inflammatory inhibition and pronounced intestinal wall.

The *abdominal CT scan* (Figure 1A–C) was performed on suspicion of diverticulitis before the transfer, which had revealed an extended diverticulosis. Furthermore, CT morphology demonstrated an appendicitis epiploica in the immediate subperitoneal region of the left lower abdomen with an oval fat isodense structure in the region of the sigmoid colon with surrounding inflammatory imbibition and a slightly accentuated adjacent intestinal wall.

### Suspicious diagnosis

– First episode of an uncomplicated diverticulitis of the sigmoid colon associated with an appendicitis epiploica under ongoing immunosuppression in status after orthotopic liver transplantation in 2013 because of hepatocellular carcinoma due to alcohol-induced liver cirrhosis

### Differential diagnoses

The spectrum of differential diagnoses comprised appendicitis epiploica, acute appendicitis, ulcerative colitis, diverticulitis of the (sigmoid) colon and colorectal carcinoma.

### Differential therapeutic approach

Based on an informed consent signed by the patient at the beginning of his clinical care, the indication for a conservative therapeutic approach with infusion therapy, administration of analgetics as well as “n. p. o.” (initial abstinence from food) and following restorative diet was seen. The calculated antibiotic therapy with Cefuroxime (2 × 1.5 g i. v., continued with 2 × 500 mg p. o.) and Metronidazol (3 × 500 mg i. v., continued with 3 × 400 mg p. o.) was administered under ongoing immunosuppression, finally for 7 d.

### Clinical course and outcome

During the inpatient stay, the conservative therapy led to a regression of the inflammatory parameters with accompanying clinical improvement. The patient was able to be discharged on the eighth day of hospital stay with a positive subjective well-being and well-tolerated diet.

### Further advice/prospect

A follow-up control of laboratory parameters, nutritional counselling and re-presentation in case of renewed symptoms were recommended in addition to a control of the serum level of the immunosuppressant drugs. Furthermore, a colonoscopy should be performed in four months accomplished by consequent and regular LTx follow-up investigations according to the established advices.

## Discussion

Immunosuppressants play an important role in the treatment of many diseases, especially in transplantation medicine or in the treatment of diseases with impaired or altered immune response (ulcerative colitis, Crohn’s disease etc.). They are usually used over a long period of time to prevent organ rejection and inflammation from flaring up again and to achieve disease control [1]. In addition to the positive effects, the intervention in the mediator metabolism can lead to a reduced release of prostaglandins and, thus, disrupt the body’s own defense reactions in inflammatory conditions, such as the lowering of the stimulus threshold for pain-conducting nociceptors. Due to these processes, immunosuppressants have some undesirable side effects/adverse events, as also demonstrated in this case report.

In general, a differentiation is made between the induction phase and the maintenance phase after LTx. The early phase is intended to induce acceptance of the donor organ within 30 days after transplantation. During this period, a triple combination consisting of calcineurin inhibitors, tacrolimus or, secondarily, ciclosporin A, in combination with a proliferation inhibitor and corticosteroids in higher doses is usually necessary. Corticosteroids have the longest, usually centre-specific experience, but the lowest level of evidence. The high postoperative doses of up to 1,000 mg of methylprednisolone per day are rapidly reversed to lower maintenance doses, which are stopped altogether after 3–6 months [2]. The reason for the rapid discontinuation is the decreasing compliance of the patients due to the numerous side effects such as body-stem-induced weight gain with full moon face, high blood pressure and diabetes mellitus. In addition, severe mood swings from euphoria to depression can occur [3]. The maintenance phase is intended to uphold immunosuppression, which should be reached after 6 months. This consists of immunosuppressive monotherapy or a combination of different immunosuppressants. The maintenance phase lasts for 10–15 years. After this period, it is decided individually whether the immunosuppression can be reduced or not. Most of the time, it is continued for the whole life.

The patient presented here was taking Mycophenolate mofetil (MMF) and Tacrolimus (FK506) during the maintenance phase. MMF is the morpholineothel ester of mycophenolic acid (MPA) and is rapidly converted into MPA after oral administration and absorption. MPA is a potent, reversible, uncompetitive inhibitor of inosine monophosphate dehydrogenase and acts as a selective inhibitor of T- and B-cell proliferation by blocking the production of guanosine nucleotides and interfering with the glycosylation of adhesion molecules [4]. Isolated from *Streptomyces tsukubaensis*, Tacrolimus is a macrolide with potent immunosuppressive properties. It binds to FKBP-12 and inhibits calcineurin, which causes a reduction in IL-2 and Interferon-γ production and, consequently, a reduced activation of T-lymphocytes. This is followed by a lowered cellular immune response. Currently, there is no consensus regarding the best immunosuppressive combination after LTx [5]. However, Tacrolimus has rapidly become the agent of choice because its efficacy and safety have been demonstrated in many studies [4]. But it also has some significant side effects, such as

– renal failure,
– neurotoxicity,
– changes in blood glucose (and)
– susceptibility to infections (or even)
– neoplasms [4].

To improve the outcome, some combinations of immunosuppressants due to the synergistic effect of their different action mechanisms have been tried. The patient presented here received one of the common combinations. MMF reinforces the action of FK506, so that the required dose can be reduced [5]. Tustumi et al. [5] have commented that this combination could reduce the need for corticosteroids, whose long-term side effects, such as

– diabetes,
– hypertension (and)
– hypercholesterolemia

are deleterious. The authors have revealed that patients, who are using this combination, seem to have a lower risk of acute rejection than those using Tacrolimus alone [5]. This statement was confirmed by Eckhoff et al. [4].

Apart from the side effects, immunosuppressants interact with other drugs. Tacrolimus is metabolised by the CYP3A4 system in the liver [6], therefore inhibitors and inducers of this system and the metabolism of FK506 may be affected. Based on the general recommendation, regular monitoring of liver and kidney parameters and determination of the levels of immunosuppressants should be performed. In case of other drugs, the correct intake should be considered with regard to side effects and interactions.

All interventions of immunosuppressants mentioned above can also lead to masking of symptoms, which can be considered a further major disadvantage of immunosuppression. For this reason, diverticulitis is often recognised too late in immunosuppressed patients as also A. Brandl et al. reported [7]. They have suggested that morbidity, such as free peritoneal perforation or complicated disease, and mortality in immunosuppressed patients with diverticulitis is significantly higher than in immunocompetent patients and, thus, the early timing of diagnosis, including CT scan, and also treatment considering elective sigmoid resection for patients with former episodes of diverticulitis is very important for a positive outcome. In general, immunosuppression and steroid intake are common risk factors for perforated diverticulitis. Therefore, he has pointed out that common guidelines for immunocompetent patients may not apply for immunosuppressed patients and the decision for elective sigmoid resection to prevent fatal outcomes due to sigmoid diverticulitis must be made individually based on additional risk factors and on an ideal time point for intervention [7].

In the case presented here, abdominal CT scan was performed very early. The scan demonstrated an extended diverticulosis and an appendicitis epiploica in the immediate subperitoneal region of the left lower abdomen with an oval fat isodense structure in the region of the sigmoid colon with surrounding inflammatory imbibition and pronounced intestinal wall.

However, abdominal ultrasound might have also helped appropriately – it is considered and established a basic diagnostic tool in case of diagnostic need for the suspicious diagnosis “unclear abdomen” or even “acute abdomen” but in experienced hands since a sufficient ultrasound-based investigation of the colon predicts developed expertise.

The appendices epiploica are fatty tissue-filled outpouchings of the subserosal connective tissue, predominantly along the taenia libera. Overall, 100–150 fatty tissue appendices can occur – clustered in the region of the sigmoid colon and transverse colon. These appendages can be inflamed by torsion or infarction. It can cause a severe abdominal pain, mainly during movement and increased breathing. The diagnosis is made on the basis of medical history and clinic findings as well as detected and confirmed by ultrasound and/or CT/MRI. It can lead to an acute abdomen, especially in the case of an ischaemic course or if the peritoneum is affected. Treatment options include conservative therapy with analgesics as well as surgical therapy like the excision of the affected appendix [8] if there is no other option or no sufficient improvement by the conservative approach. Appendicitis, cholecystitis and diverticulitis must be clarified as differential diagnoses. Acute epiploic appendicitis is a more frequent condition than case numbers would suggest and it is often misdiagnosed as diverticulitis like this case report implies [9], [10], [11], [12]. Although the CT scan showed an appendicitis epiploica, the main diagnosis was the first episode of an uncomplicated diverticulitis of the sigmoid colon. Of course, this diagnosis is also reasonable because underlying diverticulosis of the sigmoid colon was seen in the CT scan as well.

Both pathologies can be treated with conservative therapy like analgesics. In case of sigmoid diverticulitis in stage I-IIc, an antibiotic therapy is also administered (in particular, due to additional risk factors such as the status after organ transplantation and ongoing immunosuppressive medication despite the fact that no antibiotics are ussually needed in stage I according to the S3 guidelines), which is often not needed in the treatment of appendicitis epiploica because it is a self-limiting disease [13]. This patient was treated with a combination of Cefuroxime and Clont, which resulted in clinical improvement of symptomatology and lowering of the formerly elevated inflammatory laboratory level of CRP.

Cases like this are not that uncommon but they are not aware and registered yet in an appropriate manner. According to the literature available to Kremenevski et al. [9–12], paradoxical disease reactions are one of the most important chronic inflammatory systemic diseases. They defined a paradoxical disease reaction as an exacerbation or new occurrence of non-infectious inflammatory skin and other organ changes as occurred in the presented case with the extended diverticulosis and appendicitis epiploica using a substance that is therapeutically effective for the status after LTx, such as MMF and Tacrolimus. In addition, they described the occurrence of these paradoxical reactions under the therapy with biologicals as an interdisciplinary problem that must be differentiated from infectious, neoplastic and other autoimmunological diseases. As therapeutic options, the authors mentioned local therapy, symptomatic therapy, prednisolone treatment or discontinuation/reversal of the biologicals [14].

Interestingly, there were already several patients identified in the reporting department, who developed acute infections under anti-inflammatory or immunosuppressive therapy. For instance,

– a 52-year old man with ulcerative colitis under immunosuppressive therapy with Tofacitinib, a JAK inhibitor, developed acute appendicitis;
– furthermore, a 70-years old woman, who also took Tofacitinib because of rheumatoid arthritis developed acute diverticulitis of the sigmoid colon (and)
– a 54-years old female was diagnosed with diverticulitis of the sigmoid colon (stage, CDD 1b) – the medical history was significant for status after lung transplantation almost three years before because of COPD (grade IV).

In order to improve the care of patients undergoing immunosuppressive therapy and to detect such infections at an early stage, cases like these should raise attention. There are already some studies focusing on the pathomechanism of the side effects of immunosuppressants, such as “The safety of antirheumatic drugs” by Mucke et al. [15].

Unfortunately, a standard protocol for immunotherapy does not exist yet but some studies have demonstrated that the combination of MMF and Tacrolimus can reduce the dose of the calcineurin inhibitor, so the number of side effects and also the use of Corticosteroids can be decreased.

In general, it is very important for immunosuppressed patients to start diagnostics early in case of unclear symptoms in order to achieve a better and favourable outcome. In addition, the profile of side effects and interactions of the immunosuppressive medication should be known and, in addition, the patient should be informed in detail about the consequences of the therapy, such as need for regular blood checks, in particular, serum levels of the immunosuppressant agents, reduced immune system, etc.

Due to SARS-CoV-2 and the influence of immunosuppressive therapy on vaccination, the topic has become even more important and should be further discussed.

Last but not least, the role of the microbiome in a susceptible mucosa characterized by possible changes under immunosuppressive therapy, in particular, an altered immunoreactivity and immune response even at the very local site need to be considered in this complex etiopathogenesis. However, data are very sparse.

## Conclusions

The case presented is either one of several side effects of Mycophenolate mofetil and/or Tacrolimus listed in the package insert as “colonic inflammation” or “gastrointestinal inflammation”, or a described inflammatory reaction of a susceptible (gastro)intestinal mucosa or entire intestinal wall to microbes or microbial particles or agents caused by the transplantation-related immunosuppressive medication. However, diverticulitis might have also occurred accidentally in the presented 68-year old patient.

In case of suspected diverticulitis or inflammations affecting the gastrointestinal tract in general, it is important to consider the increased risk of perforation and the increased possibility of surgical complications in order to act appropriately.

The presented patient course demonstrates that conservative treatment was sufficient as a therapeutic approach even for high-risk patients as after former orthotopic LTx if the diagnosis is made in time and is based on a competent diagnostic spectrum including appropriate CT scan excluding serious complication(s). Thus, a longer inpatient stay because of a need for surgery and follow-up treatment necessities can be avoided.

## Supplementary Material

Supplementary Material
