# Supplementary material for: Paradox inflammatory reaction such as appendicitis epiploica and diverticulitis of the sigmoid colon under ongoing immunosuppression after previous liver transplantation (LTx)
Source: Innov Surg Sci. 2023 Sep 15;8(2):123–8. doi: 10.1515/iss-2023-0038 (PMC10696943; doi:10.1515/iss-2023-0038)
Supplement: Supplementary file 1 — Supplementary Material [file j_iss-2023-0038_suppl_001.pdf]

Reviewer Assessment

I. Trautwein et al.: Paradox inflammatory reaction such as appendicitis epiploica and diverticulitis of the sigmoid colon under ongoing immuno-suppression after previous liver transplantation (LTx)

Reviewers' Comments to Original Submission

Reviewer 1: Jaensch, Robert

Date received: 28-May-2023
Reviewer recommendation: **Return to author for minor modifications**
Reviewer overall scoring: **Medium**

Comments to author:
It would be better to use the names of the active substances of the drug and not the trade namens. Right now it is mixed.

The role of ultrasound as a diagnostic tool in divertikulitis is not mentioned unfortunately.

It is written that antibiotics are administered in sigma-diverticulitis Type I-IIc but according to the S3-guideline antibiotic therapy is not always required for Type I a/b.

And optionally: Maybe the role of the microbiome and possible changes under immuno supressive therapie can be addressed in the discussion. Data are very sparse.

Reviewer 2: Kraus, Armin

Date received: 07-Jun-2023
Reviewer recommendation: **Return to author for minor modifications**
Reviewer overall scoring: **Medium**

Comments to author:
Review for manuscript „Paradox inflammatory reaction such as appendicitis epiploica and diverticulitis of the sigmoid colon under ongoing immunosuppression after previous liver transplantation (LTx)"

Authors present a case report on a subject that the surgical community should be aware of, and which is worth being published.
To improve the work, I would like to remark the following:
Authors could explain a little more clearer why they are sure diverticulitis is induced by immunosuppression in this case- diverticulitis might have also occurred accidentally in a 68-year old person.
Conclusion could be shortened and the crucial points of the take-home message should be worked out more clearly. What is the one-sentence take-home message of this work?
I recommend acceptance after minor revision.

Authors' Response to Reviewer Comments

Date received: 25-Jun-2023

Reviewer 1:

I.) First reviewer's recommendation was: It would be better to use the names of the active substances of the drug and not the trade namens. Right now it is mixed.

RESPONSE: The authors followed the reviewer's instruction - see the text:

In particular, from "Januvia" to "Sitagliptin", from "Salofalk" to "Mesalazin", from "Pantozol" to "Omeprazol" and from "Delix" to "Hydrochlorothiazid" (see also "Abstract" and "Case report" section).

II.) The reviewer wondered: The role of ultrasound as a diagnostic tool in divertikulitis is not mentioned unfortunately.

RESPONSE: The reviewer is basically right. However, a sufficient ultrasound-based investigation of the colon predicts developed expertise. In addition, abdominal ultrasound is considered and established a basic diagnostic tool in case of diagnostic need for the suspicious diagnosis "unclear abdomen" or even "acute abdomen".

The authors solved it (to add "ultrasound" in the text - "Discussion" section) as follows:

" ... and pronounced intestinal wall.

However, abdominal ultrasound might have also helped appropriately - it is considered and established a basic diagnostic tool in case of diagnostic need for the suspicious diagnosis of "unclear abdomen" or even "acute abdomen" but in experienced hands since a sufficient ultrasound-based investigation of the colon predicts developed expertise.

The appendices epiploicae are ... "

III.) The reviewer's question was: It is written, that antibiotics are administered in sigma-diverticulitis Type I-IIc but according to the S3-guideline antibiotic therapy is not always required for Type I a/b.

RESPONSE: The reviewer is right - to address this aspect, the authors added the following phrase int the Text of the „"Discussion" section: " ... sigmoid diverticulitis in stage I-IIc, an antibiotic therapy is also administered (in particular, due to additional risk factors such as the status after organ transplantation and ongoing immunosuppressive medication despite the fact that no antibitoiucs are usually needed in stage I according to the S3 guidelines), which is often not needed in the treatment of appendicitis epiploicae ... "

IV.) The reviewer suggested: And optionally: Maybe a the role of the microbiome and possible changes under immuno supressive therapie can be addressed in the discussion. Data are very sparse.

RESPONSE: Thanks for this treasonable aspect, which need to be added - as follows at the end of the "Discussion" section:

" … more important and should be further discussed.

Last but not least, the role of the microbiome in a susceptible mucosa characterized by possible changes under immunosuppressive therapy, in particular, an altered immunoreactivity and immune response even at the very local site need to be considered in this complex etiopathogenesis. However, data are very sparse.

Conclusion

The case presented ... "

Reviewer 2:

1. The reviewer stated: Authors present a case report on a subject that the surgical community should be aware of, and which is worth being published.

To improve the work, I would like to remark the following: Authors could explain a little more clearer why they are sure diverticulitis is induced by immunosuppression in this case- diverticulitis might have also occurred accidentally in a 68-year old person.

RESPONSE: The reviewer is completely right - there is also the option that "diverticulitis might have also occurred accidentally in a 68-year old person" (however, in case of ongoing immunosuppressive medication).

And, no, the authors are not sure that diverticulitis is induced by immunosuppression in this case - it is a possible option of etiopathogenesis the authors want to suggest.

To address this basic option, this aspect was included in the manuscript text as follows:

„ ... Conclusion

The case presented is either one of several side effects of Mycophenolate mofetil and/or Tacrolimus listed in the package insert as "colonic inflammation" or "gastrointestinal inflammation", or a described inflammatory reaction of a susceptible (gastro)intestinal mucosa or entire intestinal wall to microbes or microbial particles or agents caused by the transplantation-related immunosuppressive medication. However, diverticulitis might have also occurred accidentally in a 68-year old patient. ...“

2. The reviewer recommended: Conclusion could be shortened and the crucial points of the take-home message should be worked out more clearly. What is the one-sentence take-home message of this work?

RESPONSE: The authors followed the reviewer’s suggestion and shortened the “Conclusion“ section by 50 % (see above) emphasizing the first paragraph coming very close to the reviewer’s specific orientation onto a “one-sentence take-home message of this work“.

Reviewers’ Comments to Revised Submission

Reviewer 1: Jaensch, Robert

Date received: 06-Jul-2023
Reviewer recommendation: **Accept in present form**
Reviewer overall scoring: **High**

Comments to author:

Suggestions have been implemented nicely.

Reviewer 2: Kraus, Armin

Date received: 07-Jul-2023
Reviewer recommendation: **Accept in present form**
Reviewer overall scoring: **High**

Comments to author:

All my comments and suggestions were implemented to my full satisfaction. I recommend acceptance in the present form.
